# Gleanings from Our Exchanges

**Published:** 1873-12-01

**Authors:** 


Extraction of Renal Calculus More than a Century Ago.—In a rather scarce book called "Mem., Maxims, and Memoirs, by William Wadd, Esq., F.L.S., Surgeon Extraordinary to the King, London, 1827," I find, on page 21, the following note or memorandum:

"Mr. Paul, a surgeon at Stroud, in Gloucestershire, lately extracted from the kidneys of a woman, by an incision through her back, a rough stone as large as a pigeon's egg, and made an entire cure. It is the first of the kind ever performed in this kingdom. *Gen's Magazine*, Aug., 1733."

This struck me as so extraordinary, and, in view of some recent cases and articles in our medical journals, so interesting, that I thought perhaps you might think it worth a place in your journal. Should you desire it, I might send you some other curious reminiscences from this same book.—Pooley.—*N. Y. Rec.*, Oct. 15, '73.—*The Clinic.*

A Strange Suggestion.—The *St. Louis New Era* makes the following strange suggestion. We hardly think it will be carried into effect. It would be a fatal advertisement for some M.D.'s: "In marriage notices it is usual to give the name of the clergyman who performed the ceremony; and with usual propriety, in obituary notices, the name of the attending physician should be given."—*The Doctor*, Nov. 1, 1873.

ON THE RADICAL CURE OF HERNIA.—Mr. Bryant, Surgeon to Guy's Hospital, believes that where a hernia can be kept up by a truss, and the patient is likely to remain in a civilized country where trusses can be obtained, any operation for the radical cure is unjustifiable. To risk the life of the patient on the theory of a cure, with the probability that he will be rendered less liable to its descent, when a truss has to be worn subsequent to the operation as a matter of safety, is, he says, a practical delusion.—*The Practice of Surgery; a Manual.* London. 1872.

### Gleanings from Our Exchanges.

CLINICAL LECTURE ON PUERPERAL CONVULSIONS.—BY PROF. W. T. LUSK, M.D.—In cases of puerperal convulsions, sometimes prodromata warn us of the impending explosion, but often enough they are entirely absent; so that, whether they exist or not, it is well to occasionally examine the urine of a pregnant woman.

When any of the ordinary prodromata are present, such as œdema of the feet and hands, puffiness of the face, singing in the ears, dimness of vision, and, what is very suspicious, the existence of certain inveterate neuralgic ailments, and at the same time albumen is detected in the urine, a shrewd guess may be hazarded that the woman will have convulsions. But even with these conditions convulsions do not necessarily take place. However, it is well to accept the warnings, and place the woman upon such treatment as shall tend to prevent the development of eclampsia. The best means for the accomplishment of this end consists in most cases in the employment of remedies likely to improve the condition of the blood, which is usually watery and much impaired in quality. Among our most reliable agents, in addition to good food, are the muriated tincture of iron and the chlorate of potash. Milk should enter largely into the diet, both on account of its nutritive properties and its special action upon the kidneys. The skin should be maintained in a healthy condition by warm baths. Diuretics and hydragogue cathartics are indicated only when the diminution in the quantity of the urine, or the lowering of the specific gravity, threatens the patient with an immediate pressing danger.

As exceptions to the foregoing directions are those cases where the women are plethoric rather than hydremic. In these, a reducing diet, saline cathartics, and sometimes even venesection to a moderate extent, may be resorted to. Formerly, all women were bled during pregnancy; and many an old woman will tell you now-a-days that in her time, when bleeding was the rule, they never had puerperal fever, or any of the dreaded accidents of the present day. Now we have passed to the opposite extreme. Cazeaux has convinced us all that the condition of a pregnant woman is either that of anemia or chlorosis. There is, however, some doubt, in spite of the chemists, whether this rule is sweepingly correct. In some primiparæ gestation appears to occasion an enormous increase of appetite; and I confess that I have at times found it impossible, in the absence of any physical signs, to associate with the rosy lips and brilliant coloring of many of these patients any idea of anemia. In such cases, should there be manifested any marked evidences of cerebral hyperemia, I should strongly incline toward the employment of the lanbet, for the sake of the immediate relief venesection occasions.

That bleeding is not necessarily the fatal operation which it is assumed to be to-day, is shown by the case reported by Cavalli, of Maria Besana, who was bled in twenty-eight years three thousand five hundred times.

The treatment of the convulsions proper depends somewhat upon our views concerning their origin.

The popular belief undoubtedly is, that they are due to the accumulation of urea in the organism, and that the presence of albumen in the urine during the seizure is to be accepted as the evidence that such an accumulation has been in progress. In connection with this theory, however, the following facts are to be borne in mind: 1. A considerable number of observations where the urine presented no traces of albumen, either before or after the convulsions. 2. A still larger collection of cases in which the albumen appeared subsequent to, but not previous to, the outbreak. The observation of this peculiarity is often ascribed to Braxton Hicks, though, as a matter of fact, his paper published in the "Obstetrical Transactions" of 1867 simply shows that he was at the time ignorant of the extent to which the matter had already been discussed in Germany. 3. When the kidneys are examined, after death, the changes found in them are usually very trivial. 4. Bright's disease existing before pregnancy is far from giving occasion to an unfavorable prognosis. I know one lady who had Bright's disease previous to her marriage, and has now had four children, yet she has never had convulsions. Another lady had one child after the disease of the kidneys had reached an advanced stage, yet there were no convulsions, though she died from the kidney affection about eighteen months afterward. Seyfert found that out of eighty pregnant women suffering from confirmed Bright's disease, only two had convulsions. 5. Patients suffering from carcinoma uteri may die from hæmorrhage, septicemia, marasmus, peritonitis, etc.; but in many cases the cancerous infiltration invades the bladder and produces occlusion of the ureters, so that death results from suppression of the urine. Many cases of death from this cause, among them fifty-four reported by Cornil and Ranvier, stand recorded as having terminated without convulsions. Each new case of this description is usually reported with a certain species of surprise by the attending physician.

Notwithstanding these facts, however, I am not to be understood as altogether rejecting the uremic theory. Clinically it remains true that albumen in the urine during pregnancy is a frequent precursor of convulsions. Often I have found, in cases of eclampsia to which I have been summoned, a total suspension at the time of the urinary secretion. Not long since I saw a patient who had already had three convulsions, and was then in the fourth. As the head was low down on the perineum, I determined to use the forceps and deliver at once. On introducing the catheter as a preliminary, I drew off three pints of retained urine from the bladder. After that the convulsions ceased. In order that uremic phenomena should be produced, it is necessary that the accumulation of urea in the tissues and fluids of the body should have gone on to a certain non-determinable extent. In cases where little food is taken, and the patient is quiet in bed, the production of urea may be very small, so that in the patients suffering from the cancerous affections above noted, death may take place from the pain caused by the distension of the ureters before a sufficient amount of urea is stored up in the system to give rise to uremic symptoms.

Certainly all cases, however, are not due to blood-poisoning. In some women the pregnant condition induces an undue excitability of the nervous system. All the tissues of the body are depreciated, and the nervous system acquires an excessive reflex susceptibility. Pressure upon the peripheral nerves during labor becomes the direct exciting case of the explosion.

Traube and Rosenstein look upon puerperal convulsions as due to anemia of the brain. The first step in the production of this condition is an hydremic state of the blood. An increased pressure in the capillaries of the brain arises from hypertrophy of the heart, which is a constant acompaniment of pregnancy, and the interference with the venous circulation due to the gravid uterus. Œdema of the brain results, and as the skull is unyielding, secondarily the vessels of the brain suffer compression, and the consequent anemia gives rise to convulsions.

On the other hand, plethoric women do have convulsions, which perhaps are due to congestion of the spinal cord.

Probably we are nearest the truth when we adopt the view that causes of puerperal convulsion are multiform. When we come to treatment, we find that, even on theoretical grounds, beneficial results are to be expected in many instances from venesection. In uremia, bleeding helps to unload the system temporarily of a part of its poison; in plethoric persons it relieves the congestion; in threatened œdema of the brain it may ward off permanent damage to the cerebral organ. In nervous anemic persons, where the origin is due to reflex excitability, it can only aggravate the disorder, and exposes such women afterward to peritoneal inflammations. Its virtue lies, it is to be remembered, however, purely in the fact that in the face of an immediate threatening danger a little time may be gained by its employment. Therefore, unless my patient is not already greatly depressed, I do not hesitate to draw from the arm twelve to sixteen ounces of blood.

Then I resort to anæsthesia, pushed to an extent sufficient not only to destroy sensibility, but likewise to overpower the voluntary motor-nerves of the body. Hypodermic injections of morphia (gr. $\frac{1}{8}$, repeated, if required once or twice) support the action of the anæsthetic, and render much less of the agent necessary. Anæsthetics and narcotics, I am sure from experience, often succeed, after moderate venesection, where previously they had failed.

But anæsthesia must must not be maintained indefinitely. When kept up for from twenty-four to forty-eight hours, it is pretty certain that the child will fall a victim to such prolonged employment. It therefore becomes incumbent to empty the uterus as soon as practicable. If labor has somewhat progressed, this is to be accomplished by forceps or version, according to the nature of the case. In the earlier stages, the Barnes dilators render us excellent service. If the cervix is too narrow to admit the smallest size, I begin with a sponge-tent. Manual dilatation is at all times a detestable practice. After dilatation the cervix is often œdematous, and then, if the head has not descended, forceps are to be used with the greatest caution.

After the labor is over, if the convulsions continue, they may be controlled by hypodermic injections of morphia. Chloroform, after confinement, is no longer a safe agent.

The patient * whose delivery you witnessed has, since her confinement, been put upon the following mixture:

℞.—Infusi. digitalis, ℥ iv.

Potassæ acetat., ℥ j.

Solv.

Tablespoonful every four hours. Her kidneys are now acting freely.—*The Medical Record*, Oct., 1873.

On the Prevention of Uterine Inflammation. — By Edward J. Tilt, M.D. — The author gave it as an admitted fact that the most frequent cause of uterine inflammation was to be found in parturition and in abortion; and his own experience led him to believe that a tedious labor and a bad miscarriage could hardly occur without entailing more or less of uterine inflammation; frequently overlooked in its onset by the medical attendant, metritis in one form or another being the almost inevitable sequel of such cases, although many years might elapse before the disease was recognized. The author proceeded to answer the following questions: 1. What are the symptoms of a bad getting up? 2. What are the organic lesions of a bad getting up that lead to uterine inflammation? 3. How to prevent a natural function from becoming a frequent cause of metritis? 1. After tracing the symptoms of a bad getting up, the author deprecated the little attention paid to the persistence of a red or muco-purulent vaginal discharge for a month or more after parturition. He wished such cases to be carefully inquired into, instead of being treated in a hap-hazard fashion by tonics and change of air. 2. Although a natural function, parturition had too often

* History of case omitted.

untoward results, such as defective uterine involution, placental ulceration of the womb, contusion and laceration of the cervix. Laceration of the cervix was represented as very common, particularly after tedious and instrumental labors. The healing by first intention of these lacerations was given as the rule when they were not extensive, and when women were healthy; but if, on the contrary, these lacerations were extensive, they did not heal in sickly women, and had originated some of the worst cases of uterine inflammation that the author had seen. Under similar unfavorable circumstances of health, the bruising of the cervix by a tedious labor was represented as beyond the power of the womb to repair, unless by the repair of ulceration thus produced. Ulceration of that part of the womb to which the placenta had been attached was considered a rare disease, sometimes following the forcible tearing away of the placenta from the wound, and originating one form of internal metritis characterized by frequent flooding. The most important and most frequent cause of uterine inflammation, and of other diseases of the womb, was said to be defective uterine involution. To an exaggerated belief in the safety of a natural function was ascribed the fact that medical men too often neglected to ascertain accurately what were the organic lesions that impeded a patient's recovery after parturition; so that, as a rule, defective involution was only recognized when time had confirmed and made it more difficult to cure. 3. The measures calculated to prevent parturition being a frequent source of metritis, were represented to be the logical deduction of the right appreciation of the damage done to the womb by parturition; and it was strongly urged that when, at the end of four or five weeks after parturition, notwithstanding fair nursing, food, wine, and tonics, women still continued weak, with persistent back-pain and muco-purulent or red vaginal discharges, instead of blindly trusting to nature, it would be wiser to ascertain, by an accurate examination, whether the inability to recover health did not depend on one of those organic lesions that could not be cured without the calling in of surgery in aid of nature. The same line of conduct was advised when women were recovering from parturition who had previously suffered from uterine disease, on account of its liability to relapse. The unusual severity of uterine inflammation that originated in abortion was said to depend on the absence of definite rules of conduct to be observed by women after miscarriage, and on the little care they then took of themselves; whereas Dr. Tilt wished the profession could persuade the public that a month of convalescence was not too much to exact after a moderate bad miscarriage; and that if, at the end of that time, a patient did not recover strength, could not walk, had pelvic pains, and a red or muco-purulent vaginal discharge, the cause of these symptoms should be carefully investigated. The author stated the difficulty of curing defective uterine involution to be in direct proportion to the time it had already lasted; and he therefore urged its speedy recognition. He recommended leeching the cervix if there were signs of active congestion of the womb, the internal administration of ergot and of iodide of potassium, the painting of the lower part of the abdomen with oleate of mercury, and vaginal injections. It was also admitted that pregnancy had sometimes cured the mischief done by a previous one.

Dr. Tilt concluded by emphatically asserting that, by a judicious management of lying-in women, and of those recovering from abortion, uterine irritation and congestion would be reduced, and lacerations healed; and that uterine inflammation would be checked in its origin, and, at all events, its acuteness and duration would be greatly diminished.

Dr. Steele (Liverpool) doubted the utility of vaginal injections as curative agents in inflammation within the cavity of the cervix or uterus, which could only be successfully combated by medication at the seat of the disease. He also thought there would be some difficulty in so localizing internal metritis as to justify the term placental ulceration.

Dr. Thomson (Edinburgh) believed that subinvolution was a frequent cause of uterine ailment.

Mr. Bracey (Birmingham) endorsed many of the views expressed in the paper, which he regarded as a most valuable communication. He understood that vaginal examination was recommended only when convalescence did not proceed favorably.

Substitution of Goat's Milk from the Living Animal for the Sucking-Bottle.—Dr. H. MacCormac maintains that goat's milk may be abstracted from the living animal, and transferred at once to the infant's stomach, by means of a tube provided with an artificial nipple. In this way it is thought the mortality hitherto attendant on artificial lactation may be greatly diminished.—*Boston Medical Journal*, Oct. 23, 1873.

According to French papers, M. Nelaton has left 7,000,000 francs, or £280,000. He had private means, and amassed a considerable sum of money by his profession.

Vaginal Ovariotomy.—Dr. Gilmore, of New Orleans, reports in a letter to Dr. Thomas, of New York, a successful case of vaginal ovariotomy performed on the wife of a physician, with the following conclusion:

Peaslee does your operation great injustice when he says it is not less hazardous than abdominal section. The vaginal tissues are possessed of a high degree of vitality, and there is not in the line of incision either fat or tendinous structures that are lowly organized to interfere with immediate union. Then, again, there are no muscles in proximity, which, called into action, would interfere with union. All these obstacles to immediate union exist in the abdominal section. Indeed, I intend hereafter, in all cases that I meet, in which I suspect an unilocular cyst, and especially when it crowds down into the pelvis posterior to the uterus, to open the vagina and tap the tumor, and, if possible, to drag it through the vaginal opening. If I fail in this, I can hold in reserve the abdominal opening, and thereby free drainage would be obtained through the vagina. Peaslee objects to it on the grounds of its being an operation difficult of execution. In a deep vagina it would be: and deep vaginæ are usually found in fat women, who are bad subjects for ovariotomy. But in the great majority of women the operation is not difficult of execution. In the case I operated on I found the whole procedure extremely simple and easy. The whole operation was executed without a change of posture, and consumed only about ten minutes. Peaslee's third objection is fully answered by the fact that in my case the whole ovary was removed.—*New Orleans Medical and Surgical Journal*, Nov., 1873.

Unfortunate Case of Sneezing.—In a letter, Dr. Y. A. Winn says, "I will mention a very singular case — singular to me at least. A gentleman informs me that when prompted to sexual commerce he is seized, immediately prior to the act, with a fit of violent sneezing: not unfrequently even the *thought* of coition produces this effect. The gentleman being married, we are a loss for a satisfactory remedy. Fortunately his wife has never *apparently* noticed this peculiarity. If she should, and he did much sneezing in the presence of other ladies, even *sneezing* might be a sufficient ground for divorce.— *West'n Lancet.* —*Phil. Times*, Oct. 15, '73.

Brown-Sequard and Segiun's Journal, *The Archives of Scientific and Practical Medicine*, ceased with the November (the sixth) No.

Too much nerve-force and too high temperature for our climate.

